# Combined genomic evaluation of Merino and Dohne Merino Australian sheep populations

**DOI:** 10.1186/s12711-024-00934-2

**Published:** 2024-09-30

**Authors:** Marine Wicki, Daniel J. Brown, Phillip M. Gurman, Jérôme Raoul, Andrés Legarra, Andrew A. Swan

**Affiliations:** 1 INRAE, INP, UMR 1388 GenPhySE, 31326 Castanet-Tolosan, France; 2 Institut de l’Elevage, 31321 Castanet-Tolosan, France; 3 AGBU, A Joint Venture of NSW Department of Primary Industries and University of New-England, Armidale, Australia; 4 Council on Dairy Cattle Breeding, Bowie, MD 20716 USA

## Abstract

**Background:**

The Dohne Merino sheep was introduced to Australia from South Africa in the 1990s. It was primarily used in crosses with the Merino breed sheep to improve on attributes such as reproduction and carcass composition. Since then, this breed has continued to expand in Australia but the number of genotyped and phenotyped purebred individuals remains low, calling into question the accuracy of genomic selection. The Australian Merino, on the other hand, has a substantial reference population in a separate genomic evaluation (MERINOSELECT). Combining these resources could fast track the impact of genomic selection on the smaller breed, but the efficacy of this needs to be investigated. This study was based on a dataset of 53,663 genotypes and more than 2 million phenotypes. Its main objectives were (1) to characterize the genetic structure of Merino and Dohne Merino breeds, (2) to investigate the utility of combining their evaluations in terms of quality of predictions, and (3) to compare several methods of genetic grouping. We used the ‘LR-method’ (Linear Regression) for these assessments.

**Results:**

We found very low Fst values (below 0.048) between the different Merino lines and Dohne breed considered in our study, indicating very low genetic differentiation. Principal component analysis revealed three distinct groups, identified as purebred Merino, purebred Dohne, and crossbred animals. Considering the whole population in the reference led to the best quality of predictions and the largest increase in accuracy (from ‘LR-method’) from pedigree to genomic-based evaluations: 0.18, 0.14 and 0.16 for yearling fibre diameter (YFD), yearling greasy fleece weight (YGFW) and yearling liveweight (YWT), respectively. Combined genomic evaluations showed higher accuracies than the evaluation based on the Dohne reference only (accuracies increased by 0.16, 0.06 and 0.07 for YFD, YGFW, and YWT, respectively). For the combined genomic evaluations, metafounder models were more accurate than Unknown Parent Groups models (accuracies increased by 0.04, 0.04 and 0.06 for YFD, YGFW and YWT, respectively).

**Conclusions:**

We found promising results for the future transition of the Dohne breed from pedigree to genomic selection. A combined genomic evaluation, with the MERINOSELECT evaluation in addition to using metafounders, is expected to enhance the quality of genomic predictions for the Dohne Merino breed.

**Supplementary Information:**

The online version contains supplementary material available at 10.1186/s12711-024-00934-2.

## Background

Australia is home to one of the world’s largest sheep populations, consisting of many different breeds, some of which are themselves structured into several lines [1]. In addition, these different breeds and lines are highly interconnected, making this population even more diversified [2, 3]. Since 2005, Sheep Genetics has provided Australian Sheep Breeding Values (ASBVs) for different breeds and flocks distributed across Australia by performing three major large-scale analyses: the MERINOSELECT evaluation for Merino, and the LAMBPLAN maternal and terminal breed evaluations. Since the introduction of this evaluation system, and even more so with the advent of genomic selection, significant increases in genetic gains have been observed in these populations [4]. However, only the main breeds benefit from a sufficiently large genomic reference population (such as the Merino, Poll Dorset, White Suffolk and Border Leicester). Across-breed evaluations are a challenge to expand the use of genomic information for these breeds or the crossbred animals [5].

In the 1990s, the Australian sheep industry was facing a changing market where wool was becoming less profitable than meat [6]. This situation was particularly challenging for Merino breeders who were observing lower fertility and carcass performance in their animals compared to the terminal or maternal sheep breeders. This was mainly the result of the long-term selection of their animals for wool traits that have unfavorable genetic correlations with growth and reproduction traits [7, 8]. In response, Australian breeders recognized the potential of the Dohne Merino breed, a more meat-oriented breed that we will call “Dohne” herein, to balance the ability of their animals to adapt to new production and wool price conditions. Indeed, the Dohne breed was considered as a “complete” breed presenting high carcass and reproduction performances, as well as high wool quality and production [9]. The year 1998 saw the first importation of Dohne from South Africa to Australia, followed 2 years later, by the creation of the Australian Dohne Breeders Association (ADBA). This breed has been largely used in crossing, mainly with Merino ewes. Since then, it has grown significantly, with, for instance, “20% of the commercial breeding ewes likely to be Dohne or their crosses with Merino” in 2014 [10]. However, the Dohne breed still conducts a separate genetic evaluation involving only purebred Dohne animals, with an emphasis on growth, reproduction, and wool traits [11]. This evaluation is moreover pedigree only, since although new purebred individuals are genotyped and phenotyped each year, their number is considered still too small to lead to accurate genomic predictions. An increase in the number of genotyped individuals would be necessary to observe a useful gain in accuracy with the transition to genomic selection. However, a circular problem often encountered is that breeders may be reluctant to genotype their animals if these genotypes are not included in the analysis, meaning that they will not benefit until the genomic reference is large enough.

However, since its introduction, the level of connectedness between Dohne sheep with other major breeds, particularly with the Merino breed, has substantially increased, and studies have shown high genetic proximity between these breeds [12]. Therefore, we can consider whether the Merino reference population could support accurate genomic predictions for the Dohne breed.

Some studies have observed improved accuracy in combining genomic evaluations of genetically close populations, which increased the reference population size [13, 14]. Conversely, genomic prediction accuracy may decrease when diverse populations are combined because of a lack of relatedness between the reference and validation populations [15]. Indeed, some studies have shown that the gain in accuracy increases with the level of relatedness between the populations [16, 17]. Overall, across-breed predictions are challenging. However, sheep breeds are often closely related to each other and less structured than what we can observe in other species. In particular, the Dohne and Merino breeds have co-developed in Australia and have been selected under similar breeding objectives to meet a common market. Therefore, we expect promising results for an across-breed prediction design for the Dohne and Merino breeds.

The main objectives of this study were (1) to characterize the genetic structure of Merino and Dohne Merino breeds and their current degree of connectedness, and (2) to investigate which reference population is the most informative for both Merino and Dohne Merino breeds and their crossbreds. This second objective also implies quantifying the benefit, in terms of accuracy, for the Dohne breed to move from pedigree to genomic evaluation. A last objective will be (3) to test different methods of accounting for missing pedigree with a simplified genetic grouping on the quality of the predictions.

## Methods

### Data

Data were extracted from two Sheep Genetics databases, the database for the Dohne Merino evaluation, and the MERINOSELECT database. The first database involves flocks registered to the Australian Dohne Breeders Association, which includes data on purebred Dohne animals only. The entire database was considered in our study. The MERINOSELECT database largely contains information on Merino flocks but also includes other breeds and crossbred animals, including crosses involving the Dohne Merino and Merino breeds. These crosses are typically the result of the use of Dohne Merino sires, whether in flocks registered to the MERINOSELECT evaluation, the Australian Merino Sire Evaluation Association (AMSEA), or in the Meat and Livestock Australia (MLA) resource flock. Furthermore, this database also includes purebred Dohne Merino animals that are part of flocks registered to the AMSEA evaluation, or flocks that were formerly members of the Australian Dohne Breeders Association that have moved to the MERINOSELECT evaluation. Dohne Merino genotypes are available in both databases but are not currently included in either the MERINOSELECT or Dohne evaluations (the latter is a pedigree-based evaluation only). The genotypes were selected from the Sheep Genetics genomic database for Dohne Merino animals and a subset of MERINOSELECT animals. As in de las Heras-Saldana et al. [18], we used a subset of MERINOSELECT based on the core flocks within the evaluation, those flocks with the most complete data recording. This resulted in 26,031 Merino genotypes and a total genomic dataset of 53,663 genotypes across both breeds. The whole pedigree and phenotypes dataset of combined MERINOSELECT and Dohne Merino evaluations were then pruned with renumf90 [19] to retain the deepest pedigree from the genotyped animals. Finally, the population that we studied was composed of purebred Dohne (D), purebred Merino (M) and crossbred (C) animals between these two breeds. In turn, the Merino animals were structured in six populations [3] that we will call “lines” in the following.

Two wool traits were examined: yearling fibre diameter (YFD, µm) and yearling greasy fleece weight (YGFW, kg), as well as one weight trait, yearling live weight (YWT, kg). All three traits were recorded from 1987 to 2022 over 848 flocks. The heritabilities of these traits are respectively of 0.74, 0.57 and 0.38 [7]. The pedigree included 2,470,173 animals born between 1960 and 2022, including 2,178,952 animals recorded for at least one of these phenotypes (Table 1). It is important to note that the traits studied here are not sex limited, have been recorded in almost all individuals and are, moreover, moderately to highly heritable.

**Table 1:** Number of animals with phenotypes and animals with phenotype and genotype by trait

Trait	Animals with phenotypes born before 2021	Animals with phenotype and genotype born before 2021	Animals with phenotypes born in 2021 or after	Animals with phenotype and genotype born in 2021 or later
YFD	1,580,590	21,232	85,666	9843
YGFW	1,262,060	20,482	89,023	8812
YWT	1,666,933	32,615	114,998	11,591
Total	2,039,079	34,655	136,527	12,481

*YFD* Yearling Fibre Diameter, *YGFW* Yearling Greasy Fleece Weight, *YWT* Yearling Liveweight, *Total* Animals presenting at least one of these three phenotypes

We studied 53,663 animals genotyped with a range of medium-density SNP chips obtained from the Sheep Genetics commercial genotyping pipeline from multiple commercial genotyping companies. After applying a first round of quality control (minimum individual call rate of 0.90 and maximum heterozygosity of 0.50), these genotypes were imputed using Beagle 5 software [20] to fill in missing SNPs up to 60,410 marker density. Then a second quality control was performed with the software preGSf90 [19], rejecting SNPs with a minor allele frequency of less than 0.05 and removal of Mendelian Conflicts. This resulted in genotype data with 57,428 effective SNPs and 52,387 animals.

### Genetic structure of the population

We studied the genetic structure of the population by a Principal Components Analysis (PCA) of the genotypes, performed with the R package ‘irlba’, available at https://CRAN.R-project.org/package=irlba [21]; and by computing Wright’s fixation index F_st_ [22, 23], which reflects differentiation of populations. We computed F_st_ for all pairs of populations, i.e. the Dohne breed and the six Merino genetic lines. We used the F_st_ estimator [22, 23] as $\widehat{Fst}=\frac{\frac{1}{n}{\Sigma }_{\text{i}}\left({\left({p}_{i}^{{b}^{\prime}}-{p}_{i}^{b}\right)}^{2}\right)}{\frac{1}{n}{\Sigma }_{\text{i}}\left({p}_{i}^{{b}^{\prime}}\left(1-{p}_{i}^{b}\right)+{p}_{i}^{b}\left(1-{p}_{i}^{{b}^{\prime}}\right)\right)}$ where ${p}_{i}^{b}$ and ${p}_{i}^{{b}^{\prime}}$ are allele frequencies for marker $i$ respectively in population $b$ and ${b}{\prime}$, and $n$ is the number of loci.

The results of the PCA were used to flag our genotyped animals as purebred Merino (M), purebred Dohne (D) and crossbred (C) animals. When we colored this PCA according to the pedigree-based expected proportion of either Merino breed or Dohne breed, we could distinguish, by visual inspection, three groups of animals. Thus, we decided to assign them to Merino, Dohne or crossbred (see Additional file 1 Figure S1) depending on the first and second Principal Component coordinates according to two equations:

1 $$ PC2 - 0.40PC1 + 5 \ge 0, $$

2 $$ PC2 - 0.40PC1 + 42 \le 0, $$

All animals whose coordinates met the condition of Eq. (1) were considered as Merino, while all animals meeting Eq. (2) were considered as Dohne and animals fitting neither equation (in the middle) were considered as Crossbred.

### Reference and validation populations

We aimed to compare the quality of predictions without vs. with performance, i.e. early prediction based on pedigree or genomic prediction vs. late prediction including also own performance. In our study, we considered validation animals all genotyped and phenotyped animals born during or after 2021. Table 1 shows the number of animals phenotyped and genotyped or not; all animals were included in the ssGBLUP analyses, but phenotypes of validation animals were removed. The cut-off at 1st January 2021 removes the last 2 years of phenotypes, roughly one generation as usual [24, 25]. Table 2 shows the number of animals in the genomic reference population (genotyped and phenotyped before 2021) and validation population (genotyped and phenotyped during or after 2021).

**Table 2:** Number of animals in the genomic reference and validation population split by breed

Breed	Animals in reference population	Animals in the validation population
Merino		
YFD	19,467	8542
YGFW	18,647	7557
YWT	29, 450	9729
Total	31, 309	10,549
Crossbred		
YFD	712	392
YGFW	791	355
YWT	1870	477
Total	2009	537
Dohne		
YFD	1053	909
YGFW	1044	900
YWT	1295	1385
Total	1337	1395

*Reference population* Animals with genotype and phenotype born before 2021, *Validation population* Animals with genotype and phenotype born in 2021 or after, *YFD* Yearling Fibre Diameter, *YGFW* Yearling Greasy Fleece Weight, *YWT* Yearling Liveweight, *Total* Animals presenting at least one of these three phenotypes

The scenarios explored mimic the situation in which a large database of Merino and crosses with Dohne exists, and Dohne breeders may join this database for genomic predictions. So, it is of interest to evaluate the extra accuracy (if any) and bias for Dohne and Merino pure breeds. Several scenarios were studied for which the composition of the reference population was altered. More specifically, we ran evaluations based on “single” reference populations (only one breed) and “joint” reference populations including both breeds and the crossbreds. For instance, in the scenario “All”, we included all the phenotypes and genotypes available. Conversely, in the scenario “D” only the phenotypes and genotypes of the Dohne animals were included in analyses. We were then able to compare the accuracy of the Dohne predicted by their own reference and the accuracy with the information from the other breeds. For all the scenarios we did not alter the composition of the validation population in the evaluation although the validation was performed independently for each population Merino, Crossbred and Dohne. Thus, we were able to check the quality of “across-breed” evaluations. To determine the impact of genetic proximity between reference and validation populations on the quality of predictions, scenarios adding only crossbred animals to the “single” reference sets were compared to the whole reference set (“All”). These included combining Crossbred and Dohne animals (scenario “C+D”) and Merino and Crossbred animals (scenario “M+C”). We did not consider the scenario M+D because the Australian breeders are heavy users of crossbreeding, therefore there are crossbred animals and a reference composed of purebred Merino and Dohne merino would not exist in practice. The data structure of these scenarios is presented in Table 3.

**Table 3:** Composition of phenotyped genomic reference population in each studied scenario

Scenario	Genomic reference population (number of animals)	Validation population (number of animals)
All	M+C+D (34,665)	M+C+D (12,481)
M	M (31,309)	M+C+D (12,481)
C	C (2009)	M+C+D (12,481)
D	D (1337)	M+C+D (12,481)
M+C	M+C (33,318)	M+C+D (12,481)
C+D	C+D (3346)	M+C+D (12,481)

*M* Merino, *C* Crossbred, *D* Dohne

### Validation

We compared the different models and scenarios using the LR method [26]. For each scenario, we removed the phenotypic and genotypic information of animals not relevant to the comparisons in the reference. For instance, for the scenario “M”, we removed phenotypes and genotypes for Dohne and Crossbred animals born before 2021, so they would not influence validation metrics. Note, however, that records of animals born before 2021 that were phenotyped but not genotyped were retained in all the scenarios, since these animals were not included in the PCA and we were therefore unable to identify them as Merino, Crossbred or Dohne. After removing these data, we ran a first evaluation called ‘Partial’, followed by a second evaluation, called ‘Whole’, in which phenotypes of validation animals were included. The evaluation ‘Partial’ mimics the first genomic evaluation performed on the focal individuals i.e. individuals with phenotypes and genotype born in 2021 or later (e.g. as shown in Tables 1 and 2), when no phenotypic information was available for them. Therefore, for this ‘Partial’ evaluation, all phenotypes of animals in columns 4 and 5 of Table 1 were removed, but their genotypes were retained. The data sets used for “Whole” included also validation phenotypes of the respective scenario population, i.e. for “M” the phenotypes of the Merino validation population were added. As an example, the detail for a given trait (YWT) and scenario (M+C) is shown in Table 4.

**Table 4:** Description of data included in the scenario “M+C” for yearling liveweight

Subset of animals	Partial		Whole		Number of animals, for YWT
	Phenotype included	Evaluated (included in pedigree [and in genotypes])	Phenotype included	Evaluated (included in pedigree [and optionally in genotypes])	
Phenotyped, non genotyped, < 2021	Yes	Yes	Yes	Yes	1,666,933–32,615 = 1,634,318
Phenotyped, genotyped, < 2021, Merino	Yes	Yes	Yes	Yes	29,450
Phenotyped, genotyped, < 2021, Crossbred	Yes	Yes	Yes	Yes	1870
Phenotyped, genotyped, < 2021, Dohne	No	Yes	Yes	Yes	1295
Phenotyped, genotyped, ≥ 2021, Merino	No	Yes	Yes	Yes	9729
Phenotyped, genotyped, ≥ 2021, Crossbred	No	Yes	Yes	Yes	477
Phenotyped, genotyped, ≥ 2021, Dohne	No	Yes	Yes	Yes	1385

From these two evaluations we extracted ‘whole’ and ‘partial’ (respectively ${\widehat{\mathbf{u}}}_{\mathbf{w}}$ and ${\widehat{\mathbf{u}}}_{\mathbf{p}}$) EBVs and GEBVs of focal individuals. Finally, from these ‘whole’ and ‘partial’ values we computed the following LR metrics [26]:

- The difference of means ${\widehat{\Delta }}_{p}=\left(\overline{{\widehat{\mathbf{u}} }_{\mathbf{p}}}\right)-\left(\overline{{\widehat{\mathbf{u}} }_{\mathbf{w}}}\right)$ which is an indicator of the bias of the ‘partial’ values
- The slope of regression of ‘whole’ on ‘partial’ values, ${b}_{p}$, which is an indicator of the dispersion
- The correlation between ‘whole’ and ‘partial’ values which is an estimator of the ratio of partial to whole accuracies, i.e. $\frac{ac{c}_{p}}{ac{c}_{w}}$
- The accuracy as: $acc=\sqrt{\frac{Cov\left({\widehat{\mathbf{u}}}_{\mathbf{p}},{\widehat{\mathbf{u}}}_{\mathbf{w}}\right)}{\left(1 -\overline{F }+\overline{diag\left(\mathbf{Q}{\mathbf{Q}}^{\mathbf{^{\prime}}}\right)} -\overline{\mathbf{Q}{\mathbf{Q} }^{\mathbf{^{\prime}}}}\right){{\sigma }^{2}}_{a,\infty }}}$ for the UPG models; and $acc=\sqrt{\frac{Cov\left({\widehat{\mathbf{u}}}_{\mathbf{p}},{\widehat{\mathbf{u}}}_{\mathbf{w}}\right)}{\left(1 -\overline{F }\right){{\sigma }^{2}}_{a,\infty }}}$ for metafounder models. Both models are detailed below. $\overline{F }$ is the average pedigree inbreeding of the focal individuals and $\mathbf{Q}$ is the matrix of expected fractions of group proportions [27] and ${\sigma }_{\alpha ,\infty }^{2}$ is the genetic variance of the validation individuals. For simplicity, it was assumed that ${\sigma }_{\alpha ,\infty }^{2}={\sigma }_{\alpha }^{2}$. Because of selection,${\sigma }_{\alpha ,\infty }^{2}$ is expected to be lower than${\sigma }_{\alpha }^{2}$, although not greatly as we did not use highly selected animals, which is the case for example with dairy bulls. Consequently, we slightly underestimate the accuracy. Because this assumption is made consistently across all comparisons, it should only change the overall magnitude of the accuracies for all traits but not their ranking. The different denominator for the UPG models is because UPGs are assumed as random with covariance$\mathbf{I}{\sigma }_{a}^{2}$, whereas in metafounder models (as detailed in the following section), algebra cancels internally with the scale factor $1+\frac{\overline{diag\left({\varvec{\Gamma}}\right)}}{2}-\overline{{\varvec{\Gamma}} }$ [28].

For bias ${\Delta }_{p}$ and slope of regression ${b}_{p},$ we obtained 95% confidence intervals from the output of the linear regression. This is an approximation, as it considers that all EBVs of focal animals have the same accuracy, and also that they are unrelated. The assumption is reasonable because they all have similar amount of information (i.e. own phenotype) and because the focal individual groups are large, and mostly unrelated.

### Consideration of missing pedigree

In this work, we compared the Unknown Parent Groups (UPG) method [27], which is the method currently used in the MERINOSELECT and Dohne evaluations to consider missing pedigree, and the metafounders method [28]. Both UPGs and metafounders effects were considered as random. UPGs assume unrelated and non-inbred base populations whereas the metafounders method considers relatedness between and within founders [29], e.g. missing parents from period *t* are assumed to be related to missing parents from period *t* + *1*. The assumption of unrelated base populations in UPGs is equivalent to assuming that the individuals in the base populations come from very large, different ancestral populations. However, this lack of prior structure makes estimation of UPGs effects harder in comparison to the random, correlated nature of metafounders [30]. Furthermore, the metafounder method better models changes in genetic variance due to crosses between populations [31], and is assumed to provide better compatibility for ssGBLUP methods between genomic and pedigree information [32, 33]. For these reasons, comparing the metafounder and UPG methods for the combined evaluation of Merino and Dohne breeds was of interest.

The current MERINOSELECT evaluation defines UPGs by flock and year of birth time periods of approximately 5 years. This manner of defining UPGs was adopted in the mid 2000s to account for (at the time) large differences in genetic merit between flocks, and high levels of unknown pedigree, particularly on the dam side. This definition led to many UPGs, around 620 in the data used for this study. However, the addition of genomic information to the evaluation has resulted in a much larger proportion of known pedigree in recent years. Before, extensive breeding conditions led to a big proportion of unknown dams. Genomic information in recent years enabled to identify these missing dams. Moreover, the metafounder framework requires the construction of a **Γ** matrix of relatedness between and within metafounders. The estimation of ${\varvec{\Gamma}}$ elements requires that each metafounder is represented well enough with genomic data. For this reason, the assignment of UPGs and metafounders was redefined in this study, with the aim of reducing the number of groups and therefore, increasing the genomic information linked to each of them. Thus, the flock-level UPGs were condensed down to 6 Merino strains, and the Dohne breed. These groups were then subdivided depending on the year of birth of the animals, according to intervals of 5 years. One of the Merino lines (line “SAMM” from “South African Mutton Merino” https://www.samm.net.au/about/) spanned only a few years and hence there was no need to split into further groups. This resulted in 31 UPGs or metafounders.

Two different methods for building the **Γ** matrix were compared. The first method estimated the 31 × 31 **Γ** by generalized least squares (GLS) as described in [34]. However, this method implies that each metafounder has sufficient genomic information to accurately estimate the base allele frequencies. Some time-based groups may be linked to large numbers of genotypes while others may be linked to very few. For example, most genotyping has occurred in recent years. Thus, a second **Γ** was constructed in a similar way to the **Γ** estimated by the “Trend” method of Wicki et al. [50]. If we assume a linear increase in relationship within a closed population [35], **Γ** by trend has the following form:

$$ {{\varvec{\Gamma}}} = \left[ {\begin{array}{*{20}c} {\Gamma_{0} } & {\Gamma_{0} } & {\Gamma_{0} } & \ldots \\ {\Gamma_{0} } & {\Gamma_{0} + n2\Delta F_{\left( \gamma \right)} } & {\Gamma_{0} + n2\Delta F_{\left( \gamma \right)} } & \ldots \\ {\Gamma_{0} } & {\Gamma_{0} + n2\Delta F_{\left( \gamma \right)} } & {\Gamma_{0} + \left( {2n} \right)2\Delta F_{\left( \gamma \right)} } & \ldots \\ \ldots & \ldots & \ldots & \ldots \\ \end{array} } \right], $$

where ${\Gamma }_{0}=\frac{2}{n}\left({\sum }_{i=1,n}{\left(2{p}_{i}-1\right)}^{2}\right)$ [36] is the relationship of the ancestral metafounder (the earliest population in the breed), obtained using ancestral allele frequencies at each of the *n* markers, $\Delta {F}_{(\gamma )}$ is the average increase in metafounder-based inbreeding per year (which corresponds to half the increase in relationship in the previous generation; hence the factor of 2), and $n$ is the number of years between two consecutive metafounders. In fact, $\Delta {F}_{(\gamma )}=\Delta F\left(1-\frac{{\Gamma }_{0}}{2}\right)$. The equation can be adapted to unequal time intervals, but we did not need that.

First, we estimated the base allele frequencies for the six lines and the Dohne breed, but because the population was not uniformly split, we used the following Least Squares estimator including $\mathbf{Q}$**,** which is the matrix of pedigree line contributions for each animal. For instance, for marker *i* with genotype calls in vector ${\mathbf{m}}_{\text{i}}$:

$$ 2{\hat{\mathbf{p}}}_{{\text{i}}} = \left( {{\mathbf{Q^{\prime}Q}}} \right)^{ - 1} {\mathbf{Q^{\prime}m}}_{{\text{i}}} , $$

where $${\widehat{\mathbf{p}}}_{\text{i}}$$ contains 7 allele frequencies (6 Merino lines + Dohne) for marker *i*. Then, we estimated the average increase in pedigree inbreeding within each of the seven populations (six Merino lines plus the Dohne breed, considering individuals with both parents known only, using the software ASReml 4.2 [37], available at http://www.vsni.co.uk. We used a linear model for inbreeding, that considered a baseline inbreeding per line *b*, a “baseline” increase with year of birth that is the same across lines ($$c)$$ and an increase per year of birth within each of the seven populations ($${d}_{j}$$). This was fit with the model:

$$ f_{i} = \mathop \sum \limits_{j = 1,7} q_{i,j} b_{j} + t_{i} c + \mathop \sum \limits_{j = 1,7} t_{i} q_{i,j} d_{j} + e_{i} , $$

where $${f}_{i}$$ is total pedigree inbreeding for individual $$i$$, $${q}_{i,j}$$ is the fraction of origin $$j$$ in individual $$i$$, $${b}_{j}$$ is the baseline inbreeding of the population and $${t}_{i}$$ is the year of birth. On output, the estimate of $$c$$ was very close to 0 (actually $${10}^{-4})$$ and it was ignored, whereas the estimates of $${d}_{j}$$ varied between − 0.001 and 0.016. Then, $$\Delta F$$ per population was assigned the estimate for $${d}_{j}$$, and negative values of $$\Delta F$$ (biologically impossible in closed populations) were set to the average of positive values.

A new metafounder was used every 5 years (except for the Merino line 48 for which only one metafounder was specified). Therefore, from one metafounder to another within the same line, the relatedness increase was five times $2\Delta F$. Finally, we made the simplifying assumption that the relatedness between the different populations was constant over time and equal to the starting values e.g. for populations *k* and *l*, ${\gamma }_{k,l}$, with value ${\gamma }_{k,l}=\frac{2}{n}{\sum }_{i=1,n}\left(2{p}_{i(k)}-1\right)\left(2{p}_{i\left(l\right)}-1\right)$. Our final **Γ** had the following form, plugging in the different estimates, for each population or pair of populations, estimates of ${\Gamma }_{0}$, $\Delta F$ and ${\gamma }_{k,l}$:

$${{\varvec{\Gamma}}} = \left( {\begin{array}{*{20}c} {\Gamma_{0\left( k \right)} } & {\Gamma_{0\left( k \right)} } & {\Gamma_{0\left( k \right)} } & \ldots & {\gamma_{k,l} } & {\gamma_{k,l} } & {\gamma_{k,l} } & \ldots \\ {\Gamma_{0\left( k \right)} } & {\Gamma_{0\left( k \right)} + n2\Delta F_{{\left( {k,\gamma } \right)}} } & {\Gamma_{0\left( k \right)} + n2\Delta F_{{\left( {k,\gamma } \right)}} } & \ldots & {\gamma_{k,l} } & {\gamma_{k,l} } & {\gamma_{k,l} } & \ldots \\ {\Gamma_{0\left( k \right)} } & {\Gamma_{0\left( k \right)} + n2\Delta F_{{\left( {k,\gamma } \right)}} } & {\Gamma_{0\left( k \right)} + 2n2\Delta F_{{\left( {k,\gamma } \right)}} } & \ldots & {\gamma_{k,l} } & {\gamma_{k,l} } & {\gamma_{k,l} } & \ldots \\ \ldots & \ldots & \ldots & \ldots & \ldots & \ldots & \ldots & \ldots \\ {} & {} & {} & {} & {\Gamma_{0\left( l \right)} } & {\Gamma_{0\left( l \right)} } & {\Gamma_{0\left( l \right)} } & \ldots \\ {} & {symmetric} & {} & {} & {\Gamma_{0\left( l \right)} } & {\Gamma_{0\left( l \right)} + n2\Delta F_{{\left( {l,\gamma } \right)}} } & {\Gamma_{0\left( l \right)} + n2\Delta F_{{\left( {l,\gamma } \right)}} } & \ldots \\ {} & {} & {} & {} & {\Gamma_{0\left( l \right)} } & {\Gamma_{0\left( l \right)} + n2\Delta F_{{\left( {l,\gamma } \right)}} } & {\Gamma_{0\left( l \right)} + 2n2\Delta F_{{\left( {l,\gamma } \right)}} } & \ldots \\ {} & {} & {} & {} & {} & {} & {} & \ldots \\ \end{array} } \right),$$

The estimated ${\varvec{\Gamma}}$ matrix of this form was used in the construction of the ${\mathbf{H}}^{-{\varvec{\Gamma}}}$ matrix used in ssGBLUP as detailed below.

### Models

We ran multitrait evaluations. For YFD we applied the following model:

3 $$ {\mathbf{y}} = {\mathbf{X }} {\varvec{\upbeta}}+ {\mathbf{Z}}_{{\mathbf{a}}} {\mathbf{u}} + {\mathbf{Z}}_{{{\mathbf{sfy}}}} {\mathbf{sfy}} + {\mathbf{e}}, $$

while for YGFW and YWT, we applied the following model:

4 $$ {\mathbf{y}} = {\mathbf{X }} {\varvec{\upbeta}} + {\mathbf{Z}}_{{\mathbf{a}}} {\mathbf{u}} + {\mathbf{Z}}_{{\mathbf{m}}} {\mathbf{u}}_{{\text{m}}} + {\mathbf{Z}}_{{{\mathbf{mpe}}}} {\mathbf{mpe}} + {\mathbf{Z}}_{{{\mathbf{sfy}}}} {\mathbf{sfy}} + {\mathbf{e}}, $$

where $\mathbf{y}$ is the vector of phenotypes, ${\varvec{\upbeta}}$ is the vector of fixed effects, $\mathbf{u}$ is the vector of animal genetic effects, ${\mathbf{u}}_{\text{m}}$ is the vector of random maternal genetic effects (correlated to $\mathbf{a}$), $\mathbf{m}\mathbf{p}\mathbf{e}$ is the vector of maternal permanent environmental effects, $\mathbf{s}\mathbf{f}\mathbf{y}$ is the vector of random sire by flock-year effects, and $\mathbf{e}$ is the vector of residual effects. Maternal effects (genetic and permanent environmental) were considered for YGFW and YWT only. In fact, animal and maternal effects included UPG or metafounder effects. For UPGs $\mathbf{g}$, the variance–covariance matrix of animal genetic and maternal genetic effects is $$Var\left( {\begin{array}{*{20}c} {{\mathbf{u}}^{*} } \\ {{\mathbf{u}}_{{\text{m}}} } \\ \end{array} } \right) = {\mathbf{G}}_{0} \otimes {\mathbf{A}}$$ with $\mathbf{u}=\mathbf{Q}\mathbf{g}+{\mathbf{u}}^{\mathbf{*}}$ and similarly for ${\mathbf{u}}_{\text{m}}$, where $\mathbf{Q}$ is the matrix of expected fractions of genes coming from each group and $\mathbf{g}$ is the vector of UPG effects modelled as random, $Var\left( {\mathbf{g}} \right) = {\mathbf{G}}_{0} \otimes {\mathbf{I}}.$ For metafounders, $$Var\left( {\begin{array}{*{20}c} {{\mathbf{u}}^{*} } \\ {{\mathbf{u}}_{{\text{m}}} } \\ \end{array} } \right) = {\mathbf{G}}_{0} \otimes {\mathbf{H}}_{{{\varvec{\Gamma}}}} ,$$ where ${\mathbf{H}}_{{\varvec{\Gamma}}}$ includes the metafounder effects. Matrix ${\mathbf{G}}_{0}$ is a 5 × 5 matrix where the 3 × 3 upper left block contains the genetic covariances for the 3 traits across the direct effect, the 2 × 2 lower right block contains the genetic covariances for the 2 traits across the maternal effect, and the 3 × 2 off diagonal blocks contain the covariances across direct and maternal effects for all traits. The variance–covariance matrix of maternal permanent environmental effects is $Var\left( {{\mathbf{mpe}}} \right) = {\mathbf{M}}_{0} \otimes {\mathbf{I}},$where ${\mathbf{M}}_{0}$ is a diagonal 2 × 2 matrix that contains the maternal permanent environmental variances for the YGFW and YWT traits. The variance–covariance matrix of sire by flock-year effects is $Var\left( {{\mathbf{sfy}}} \right) = {\mathbf{S}}_{0} \otimes {\mathbf{I}},$ where ${\mathbf{S}}_{0}$ is a 2 × 2 diagonal matrix that contains the sire by flock-year variances for the YGFW and YWT traits. The variance–covariance matrix of residual effects is $Var\left( {\mathbf{e}} \right) = {\mathbf{R}}_{0} \otimes {\mathbf{I}},$ where ${\mathbf{R}}_{0}$ contains the residual covariances across the three traits.

The variance components used here are those used in the official genetic evaluations (the same components are used in the Dohne Merino and MERINOSELECT evaluations) and have been recently re-estimated [18]. **X**, ${\mathbf{Z}}_{\mathbf{a}}$, ${\mathbf{Z}}_{\mathbf{m}}$, ${\mathbf{Z}}_{\mathbf{m}\mathbf{p}\mathbf{e}}$ and ${\mathbf{Z}}_{\mathbf{s}\mathbf{f}\mathbf{y}}$ are the incidence matrices for fixed effects and random effects of animal breeding value, dam, maternal permanent environment and sire by flock-year respectively. Phenotypes were pre-adjusted for known fixed effects including age at measurement, birth-rearing type, and age of dam, so that the only fixed effect fitted was the contemporary group for each trait as done in the official evaluation [37–41].

In order to quantify the interest for the Dohne breed to move from a pedigree-base evaluation to a genomic evaluation we performed BLUP and SSGBLUP evaluations applying the two models described previously. In the case of BLUP, $$\left( {\begin{array}{*{20}c} {\mathbf{u}} \\ {{\mathbf{u}}_{{\mathbf{m}}} } \\ \end{array} } \right)\user2{ }\sim {\text{N}}\left( {\begin{array}{*{20}c} 0 \\ 0 \\ \end{array} ,{\mathbf{G}}_{0} \otimes {\mathbf{A}}} \right)$$$$,$$ where $$\mathbf{A}$$ is the relationship matrix based on pedigree information. In the case of SSGBLUP, $$\mathbf{u}$$ and $${\mathbf{u}}_{\mathbf{m}}$$ are following $${\text{N}}\left( {0,{\mathbf{G}}_{0} \otimes {\mathbf{H}}_{{{\varvec{\Gamma}}}} } \right)$$ where $${\mathbf{H}}_{{\varvec{\Gamma}}}$$ is the relationship matrix based on both genomic and pedigree information, but which differ for UPGs [42], where it was called “QP Model”

$$ {\text{H}}_{{{\text{Q}}\Sigma }}^{*} = {\text{A}}_{\Sigma }^{*} + \left[ {\begin{array}{*{20}c} 0 & 0 & 0 \\ 0 & {{\text{G}}^{ - 1} - {\text{A}}_{22}^{ - 1} } & { - \left( {{\text{G}}^{ - 1} - {\text{A}}_{22}^{ - 1} } \right){\text{Q}}_{2} } \\ 0 & { - {\text{Q}}_{2}^{\prime } \left( {{\text{G}}^{ - 1} - {\text{A}}_{22}^{ - 1} } \right)} & {{\text{Q}}_{2}^{\prime } \left( {{\text{G}}^{ - 1} - {\text{A}}_{22}^{ - 1} } \right){\text{Q}}_{2} } \\ \end{array} } \right], $$

or metafounders [42],

$$ {\text{H}}_{{\Gamma }}^{ - 1} = {\text{A}}_{{\Gamma }}^{ - 1} + \left[ {\begin{array}{*{20}c} 0 & 0 & 0 \\ 0 & {{\text{G}}_{05}^{ - 1} - {\text{A}}_{{{\Gamma }22}}^{ - 1} } & 0 \\ 0 & 0 & 0 \\ \end{array} } \right], $$

where ${\mathbf{G}}_{05}$ is the genomic relationship matrix calculated by VanRaden [43] from genotypes (using observed allele frequencies for UPG and 0.5 for metafounders) and blending the genomic and pedigree matrices with weights 0.95 and 0.05 on $\mathbf{G}$ and ${\mathbf{A}}_{22}$ (or ${\mathbf{A}}_{22({\varvec{\Gamma}})}$), respectively for invertability.

In summary, we compared 6 models:

- BLUP-UPG (BLUP with UPG)
- ssGBLUP-UPG (ssGBLUP with UPG)
- BLUP-MetaGLS (BLUP with metafounders and with **Γ** matrix estimated by GLS)
- ssGBLUP-MetaGLS (ssGBLUP with metafounders and with **Γ** matrix estimated by GLS)
- BLUP-MetaTrend (BLUP with metafounders and with **Γ** matrix estimated “by trend”)
- ssGBLUP-MetaTrend (ssGBLUP with metafounders and with **Γ** matrix estimated “by trend”)

We used the software ‘BLUP90IOD3’ [44] to perform all these evaluations.

## Results

### Genetic structure of the population

We found quite low Fst values across the seven populations (Table 5) with the highest values being 0.037 to 0.048 between the line SAMM (South African Meat Merino) and the other populations. The closer the Fst values are to 0, the less differentiated the populations so we can see here that the populations are very similar to each other. Furthermore, aside from line SAMM, the highest values did not include the Dohne breed, and there were greater differences within the Merino lines than between Dohne and Merino lines. We can even see that the two populations closest to each other were the Merino line “MerinoS”, selected particularly for body size, and the Dohne breed (Fst value of 0.002).

**Table 5:** Fst estimates between lines

Line	RF	SAMM	Merino	MerinoFM	MerinoS	MerinoUF	DM
RF		0.043	0.005	0.007	0.021	0.009	0.017
SAMM			0.037	0.040	0.048	0.041	0.046
Merino				0.003	0.013	0.004	0.010
MerinoFM					0.009	0.003	0.006
MerinoS						0.006	0.002
MerinoUF							0.004
DM							

*RF* research flocks, *SAMM* South-African Meat Merino, *MerinoFM* Merino Fine Medium, *MerinoS* Merino Strong, *MerinoUF* Merino Ultrafine, *DM* Dohne Merino breed. RF, SAMM, Merino, MerinoFM, MerinoS and Merino UF are merino lines

**Fig. 1:**
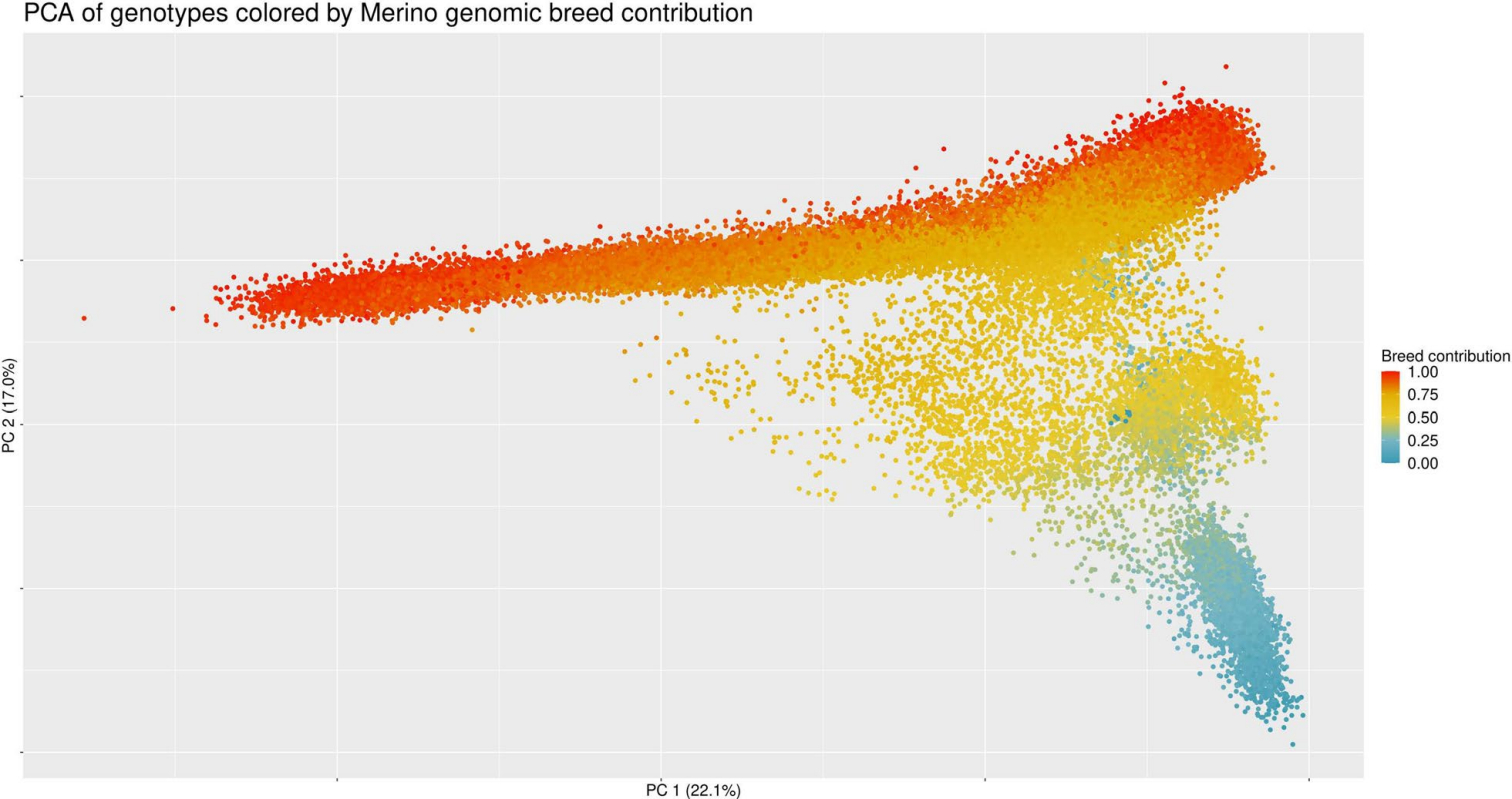
Principal Components Analysis (PCA) of the genotypes colored by Merino breed genomic contribution. *PC* Principal component

The results of the PCA of the genotypes reveal three different groups of animals, mainly split on the second Principal Component (PC2), but not clearly separated (Fig. 1). When we colored the individuals according to their respective Merino breed genomic contribution (as inferred from pedigree), we observed that the large group with highest coordinates on PC2 have a strong Merino breed contribution whereas the breed contribution decreases with lower PC2 coordinates (Fig. 1). When we colored this PCA according to the Dohne breed contribution (not shown here), we observed the opposite pattern. Thus, we considered the group on the upper part of the graph as purebred Merino animals (M), the group on the lower part as purebred Dohne animals and the group in the middle part as crossbred animals. The fact that these groups are mainly separated on PC2 (explaining 17% of the variance), and not on PC1 (22.1% of the variance) shows that most of the difference is observed within the Merino breed and not between Merino and Dohne breeds, consistent with the Fst values. This is a byproduct of the PCA trying to maximize explained differences across the whole data set, which includes many more Merino than Dohne animals.

### Metafounder relationship matrices

The ${\Gamma }_{0}$ values between the 6 Merino lines and the Dohne breed, used to build the ${\varvec{\Gamma}}$ matrix by the “Trend” method, are presented in Table 6.

**Table 6:** ${\Gamma }_{0}$ estimates between lines and average increase in pedigree inbreeding for each line and per year (from 1960 to 2022)

Line	RF	SAMM	Merino	MerinoFM	MerinoS	MerinoUF	DM	$\Delta F$
RF	0.498	0.481	0.482	0.480	0.473	0.480	0.474	0.0000
SAMM	0.481	0.595	0.481	0.479	0.480	0.478	0.478	0.0018
Merino	0.482	0.481	0.481	0.477	0.476	0.477	0.475	0.0000
MerinoFM	0.480	0.479	0.477	0.484	0.483	0.480	0.483	0.0017
MerinoS	0.473	0.480	0.476	0.483	0.511	0.489	0.503	0.0001
MerinoUF	0.480	0.478	0.477	0.480	0.489	0.487	0.489	0.0003
DM	0.474	0.478	0.475	0.483	0.503	0.489	0.502	0.0002

*RF* research flocks, *SAMM* South-African Meat Merino, *MerinoFM* Merino Fine Medium, *MerinoS* Merino Strong, *MerinoUF* Merino Ultrafine, *DM* Dohne Merino breed. RF, SAMM, Merino, MerinoFM, MerinoS and Merino UF are Merino lines

The relatedness estimates between the different ancestral metafounders were very similar to each other (off-diagonal elements): on average 0.481 with a variance very close to 0. The relatedness estimates within ancestral metafounders (diagonal elements) were slightly higher, on average 0.504, but still very similar to each other (variance of 0.001). The average increase in pedigree inbreeding within each line was equal to 0 or very close to 0 (Table 6). When the value of $\Delta F$ was 0, we replaced it with the average of non-zero values.

Finally, when we compared the two final ${\varvec{\Gamma}}$ matrices used in the models BLUP-MetaGLS and ssGBLUP-MetaGLS and BLUP-MetaTrend and ssGBLUP-MetaTrend respectively, we obtained differences ranging from − 0.068 to 1.302. Globally, all values were very similar between the two ${\varvec{\Gamma}}$ matrices except for three groups: the group relative to the SAMM line and two groups relative to the Dohne breed, for which the values with the GLS method were clearly higher than the rest of the matrix.

### Quality of genomic predictions

Overall, in terms of genomic predictions quality, all the scenarios that included Merino animals in the reference (i.e. scenarios “All”, “M” and “M+C”) obtained very similar results, which is why we only present the results of the "All" reference here. In the same way, the three BLUP models obtained very similar results, as did the two ssGBLUP models with metafounders. Thus, we present the results of models BLUP-UPG, ssGBLUP-UPG and ssGBLUP-MetaGLS only. In the following we will use the notation “validation-reference” to refer to the different scenarios. For example, the “D-All” scenario refers to the results of the Dohne validation evaluated by the “All” reference.

#### Accuracy

Genomic prediction accuracies for YFD, YGFW and YWT are presented in Tables 7, 8 and 9, respectively.

**Table 7:** Genomic prediction accuracy based on LR method, observed across reference populations, validation populations and models for yearling fibre diameter

Reference	Validation	Model		
		BLUP-UPG	ssGBLUP-UPG	ssGBLUP-MetaGLS
All	M	0.45	0.60	0.64
	C	0.34	0.57	0.62
	D	0.35	0.48	0.51
C	M	0.41	0.51	0.54
	C	0.33	0.47	0.51
	D	0.34	0.43	0.46
D	M	0.41	0.48	0.51
	C	0.32	0.44	0.47
	D	0.35	0.41	0.44
C+D	M	0.41	0.49	0.52
	C	0.34	0.46	0.51
	D	0.35	0.42	0.45

*All* Merino + Crossbred + Dohne Merino reference, *M* Merino, *C* Crossbred, *D* Dohne Merino, *BLUP-UPG* Blup model with UPG, *ssGBLUP-UPG* ssGBLUP model with UPG, *ssGBLUP-MetaGLS* ssGBLUP model with metafouders and gamma matrix built by Generalized Least Squares

**Table 8:** Genomic prediction accuracy based on LR method, observed across reference populations, validation populations and models for yearling greasy fleece weight

Reference	Validation	Model		
		BLUP-UPG	ssGBLUP-UPG	ssGBLUP-MetaGLS
All	M	0.48	0.65	0.71
	C	0.36	0.51	0.58
	D	0.38	0.52	0.55
C	M	0.45	0.51	0.54
	C	0.36	0.42	0.48
	D	0.35	0.43	0.47
D	M	0.45	0.51	0.56
	C	0.35	0.40	0.46
	D	0.38	0.46	0.50
C+D	M	0.45	0.60	0.56
	C	0.37	0.42	0.47
	D	0.38	0.51	0.51

See Table 7 for abbreviations

**Table 9:** Genomic prediction accuracy based on LR method, observed across reference populations, validation populations and models for yearling liveweight

Reference	Validation	Model		
		BLUP-UPG	ssGBLUP-UPG	ssGBLUP-MetaGLS
All	M	0.43	0.62	0.68
	C	0.47	0.74	0.66
	D	0.37	0.57	0.53
C	M	0.42	0.53	0.59
	C	0.45	0.67	0.56
	D	0.31	0.52	0.43
D	M	0.42	0.49	0.56
	C	0.45	0.54	0.56
	D	0.38	0.48	0.47
C+D	M	0.42	0.46	0.56
	C	0.46	0.58	0.56
	D	0.38	0.41	0.48

See Table 7 for abbreviations

For the trait YFD, the accuracies were slightly greater for the validation “M” than for “C” or “D” validations. On average 0.42, 0.33 and 0.35 for the validation “M”, “C” and “D” respectively for the BLUP models; and 0.60, 0.51 and 0.45 for the ssGBLUP models. For the BLUP models and within each validation, we observe very similar accuracies between the different references. However, we observed a stronger effect of the reference for the ssGBLUP models. The highest accuracies were observed for the scenarios “All” for the three validations. Similarly, the greatest gains in accuracy between the BLUP and ssGBLUP models were observed with the reference “All”. Specifically, we observe a gain in accuracy of 0.13 from BLUP-UPG to ssGBLUP-UPG for the scenario D-All whereas this gain is only of 0.06 in the scenario D-D. For this trait, the models with metafounders were more accurate than the UPG models: on average 0.48 for the ss-GBLUP-UPG against 0.52 for the ssGBLUP-MetaGLS.

Globally, in terms of accuracy, the results for the trait YGFW were very similar to the results for the trait YFD. We did not observe a major effect of the reference population on the accuracy in the case of BLUP models. The highest gain in accuracies with the addition of genomic information was observed in the scenario “All”: increase of 0.14 from BLUP-UPG to ssGBLUP-UPG for the scenario D-All against 0.08 for the scenario D-D. For this trait as well, the metafounders model was more accurate than UPG one: on average 0.50 against 0.53 for the scenario ssGBLUP-UPG and ssGBLUP-MetaGLS respectively (except for the validation “M” in the scenario “C+D” for which the UPG model was 0.04 more accurate than the metafounders models).

For the trait YWT, we typically observed the same patterns as for the two previous traits but with more variations between references or models. Still, the largest change in accuracies from BLUP to ssGBLUP models were seen in the scenario “All”: increase of 0.20 from BLUP-UPG to ssGBLUP-UPG for the scenario D-All and 0.10 for the scenario D-D. Finally, for the validation “M”, metafounders models was more accurate than the UPG models: on average 0.53 against 0.60 for the models ssGBLUP-UPG and ssGBLUP-MetaGLS respectively. However, the comparison of metafounder versus UPG models was less clear for the two other validations which, sometimes, showed higher accuracies with UPG than with metafounders.

#### Dispersion

**Fig. 2:**
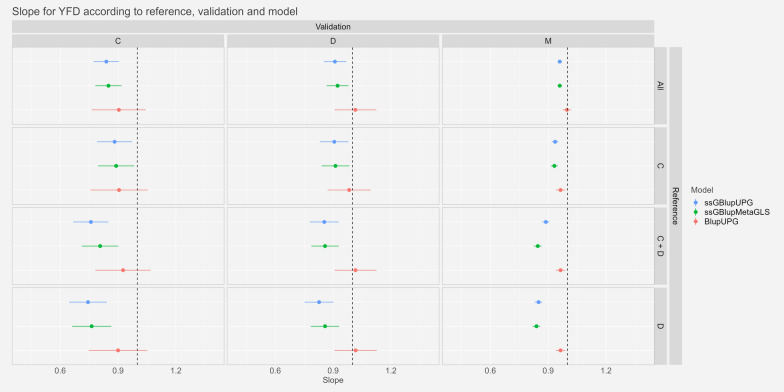
Estimates and confidence intervals of slope of regression (${\widehat{{\varvec{b}}}}_{{\varvec{p}}}$) of regression of GEBVw on GEBVp, observed across scenarios, validation populations and models for Yearling Fibre Diameter. *C* Crossbred, *D* Dohne, *M* Merino, *BlupUPG* Blup model with UPG, *ssGBlupUPG* ssGBlup model with UPG, *ssGBlupMetaGLS* ssGBlup model with metafounders and gamma matrix built by Generalized Least Squares, *ssGBlupMetaTrend* ssGBlup model with metafounders and gamma matrix built by trend

**Fig. 3:**
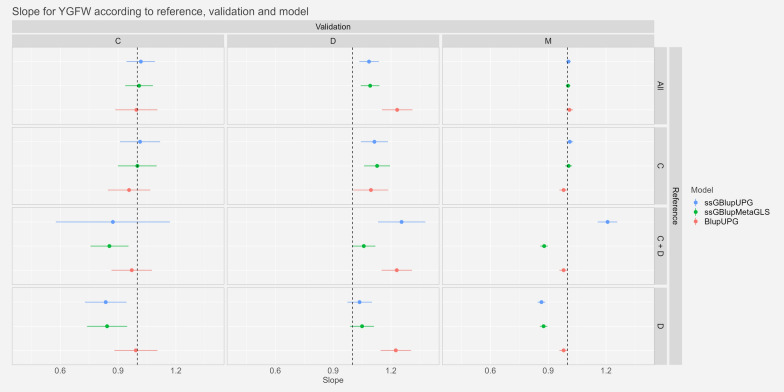
Estimates and confidence intervals of slope of regression (${\widehat{{\varvec{b}}}}_{{\varvec{p}}}$) of regression of GEBVw on GEBVp, observed across scenarios, validation populations and models for Yearling Greasy Fleece Weight. See Fig. 2 for abbreviations

**Fig. 4:**
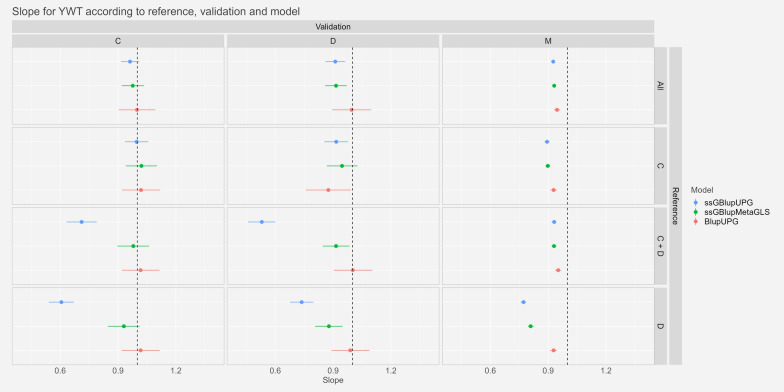
Estimates and confidence intervals of slope of regression (${\widehat{b}}_{p}$) of regression of GEBVw on GEBVp, observed across scenarios, validation populations and models for Yearling Liveweight. See Fig. 2 for abbreviations

The regression slope (${\widehat{b}}_{p}$) and associated 95% confidence intervals of “whole” GEBVs on “partial” GEBVs for YFD, YGFW and YWT are presented in Figs. 2, 3 and 4, respectively. An estimated slope less than one implies that GEBVs of candidates with genomic information only are overestimated compared to their GEBVs with phenotypes included. For the trait YFD, we observed slopes non-significantly different from one for the BLUP models and slopes slightly lower than one for the models including genomic information with confidence intervals ranging from 0.65 to 1.13. Globally, the reference “All” gave the closest slopes from one: slopes ranging from 0.95 to 1.02 for the scenario “M-All” and from 0.85 to 1.12 for the scenario “D-All”.

For the trait YGFW, we observed slopes not significantly different from one or very close to one with the references “All” and “C”. Particularly for the scenario “M-All” the confidence intervals ranged from 0.99 to 1.03 and from 1.04 to 1.31 for the scenario “D-All”. For the scenarios with references “C+D” and “D” the slopes were further from one especially for the model ssGBLUP-UPG: between 0.58 and 1.31 with the reference “C+D”.

Finally, for the trait YWT, as for YGFW, slopes were very close to the expected value of one with references “Mer” and “C”: confidence intervals between 0.92 and 0.96 for the scenario “M-All” and between 0.86 and 1.10 for the scenario “D-All”. The references “C+D” and “D” showed more variable slopes particularly the model ssGBLUP-UPG the slopes could be as low as 0.46 in the scenario “C-D”.

#### Bias

**Fig. 5:**
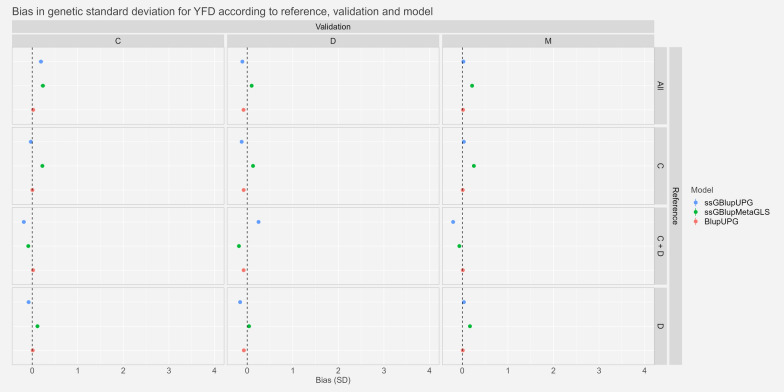
Estimates and confidence interval of bias (${\widehat{\Delta }}_{p}$) between whole and partial GEBV, observed across scenarios, validation populations and models for Yearling Fibre Diameter expressed in genetic standard deviation. See Fig. 2 for abbreviations

**Fig. 6:**
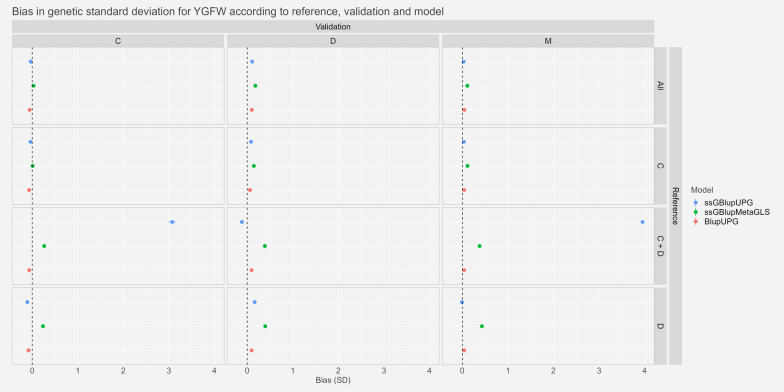
Estimates and confidence intervals of bias (${\widehat{\Delta }}_{p}$) between whole and partial GEBV, observed across scenarios, validation populations and models for Yearling Greasy Fleece Weight expressed in genetic standard deviation. See Fig. 2 for abbreviations

**Fig. 7:**
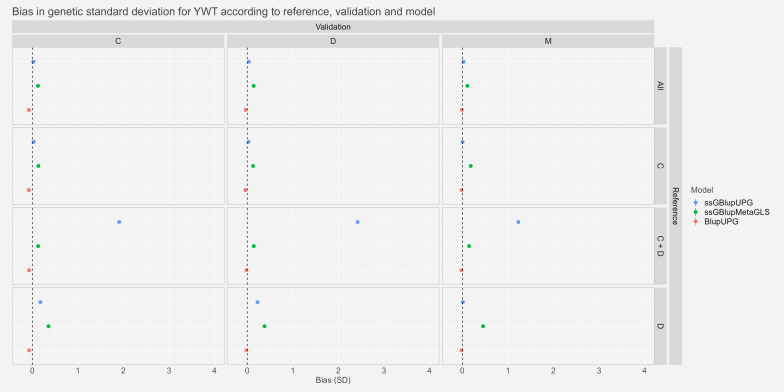
Estimates and confidence intervals of bias (${\widehat{\Delta }}_{p}$) between whole and partial GEBV, observed across scenarios, validation populations and models for Yearling Liveweight expressed in genetic standard deviation. See Fig. 2 for abbreviations

The bias estimates (${\widehat{\Delta }}_{p})$ and their associated 95% confidence intervals scaled by the genetic standard deviation for YFD, YGFW and YWT are presented in Figs. 5, 6 and 7, respectively. As a reminder, negative bias implies that GEBVs of candidates (with genomic information only) are underestimated compared to their GEBVs estimated with phenotypes. For the trait YFD, we observed very small bias with confidence intervals ranging from − 0.23 to 0.29 genetic standard deviation. Bias for the models BLUP-UPG and ssGBLUP-UPG were, in most of the cases not significantly different from zero whereas the model ssGBLUP-MetaGLS showed small bias.

For the traits YGFW and YWT, we saw very small bias or bias not significantly different from zero for the two references “All” and “C” with confidence intervals ranging from − 0.11 and 0.21genetic standard deviation for YGFW and − 0.12 and 0.19 for YWT. With the references “D” and “C+D” we saw higher biases. Especially, for the trait YGFW the model ssGBLUP-UPG reached very high biases of around 3 and 4 genetic standard deviation in scenarios “C−C+D” and “M−C+D” respectively. Similarly, for YWT, biases were around 2, 2.5 and 1 genetic standard deviation for the scenarios “C−C+D”, “D−C+D” and “M−C+D” respectively with ssGBLUP-UPG model.

## Discussion

### Genetic structure of the population

The low Fst values obtained between the Merino and Dohne breeds were in agreement with a previous study [12], which also found low genetic differentiation between these breeds. However, in this previous study, the Fst values were higher than ours: 0.06 (between the Australian Merino and Dohne breeds) compared to values ranging from 0.002 to 0.046 in our study depending on the Merino line being considered. The previous study [12] considered only 918 Merino genotypes and 30 Dohne genotypes, which was much less than the number of animals we used, and may have led to less accurate estimates. We can also speculate that recent genetic links may have contributed to increase the proximity of these breeds as well.

In the same study [12], the genetic differentiation between some Merino lines was greater than between breeds, which is also in agreement with our observations. The greatest genetic distance was between the “Strong” and “Fine-Medium” Merino lines, corresponding to the MerinoS and MerinoFM lines in Table 5. In our study, the largest distance also involved the “Strong” Merino lines but with the line “SAMM”. This can be explained by the fact that this line corresponded to the South African Meat Merino breed, originating from animals imported to South Africa from Europe in the 1930s, long after the formation of the Australian Merino in the 1800s. Nevertheless, the Fst of 0.04 between the “MerinoS” (“Strong”) line and the “MerinoFM” (“Fine-Medium”) line was one of the highest values.

Our PCA results suggested three groups of animals, one of them indicating a crossbred population, with none of the groups clearly separated from each other. This result is consistent with previous PCAs [12, 45] performed between different Australian and South African sheep breeds, which showed that the Dohne breed was the closest to the Merino breed with overlap between the two. Finally, the percentage of variance explained by the two first components (22.1 and 17%) was higher than in these previous studies [12, 45] which is consistent with greater homogeneity in our sample.

### Metafounder relationship matrices

The ancestral relatedness within each line (${\Gamma }_{i,i}$), used in the “Trend” method, showed slightly variable values between 0.481 and 0.595. These values, different from zero, do not match the assumption of unrelated pedigree founders. Similarly, the ancestral relatedness between lines (${\Gamma }_{i,j}$ with i ≠ j), greater than 0, indicates similarity of base populations. There were no large differences between the different lines, which indicates that they are quite similar genetically, consistent with the Fst findings.

For the trend in increasing metafounders group relatedness with time, we obtained values ranging from 0 to 0.0018. These very low values are consistent with the high level of crossing and the high diversity known in these lines [3, 46]. They also result in shrinkage of the metafounder solutions toward each other, within line.

Finally, for the comparison between the matrix GammaGLS (used in the models BLUP-MetaGLS and ssGBLUP-MetaGLS) and the matrix GammaTrend (used in the models BLUP-MetaTrend and ssGBLUP-MetaTrend), we note that the two groups showing the largest difference corresponded to the groups represented by the smallest number of animals. Moreover, in all the estimates obtained from the “Trend” method, all values were within the same order of magnitude, whereas with the “GLS” method these two groups clearly stand out from the rest. We can therefore assume more realistic estimates for the “Trend” method, which divided genomic information into 7 lines instead of 31 groups for the GLS method, which potentially resulted in estimation difficulties. Overall, the use of metafounders enables considering relatedness between base populations, even if the ${\varvec{\Gamma}}$ structure is not perfect. Kudinov et al. [32] found that increasing the number of metafounders improved the ${\varvec{\Gamma}}$ estimate, but we doubt that in our case, unlike theirs, the information linked to each group would have been sufficient.

### Quality of genomic predictions

We observed gains in accuracy from BLUP to ssGBLUP evaluations for all the scenarios, traits and models which was expected. Particularly, for the evaluation of the Dohne breed based on its own reference (scenario “D-D”), we observed relative gains in accuracy of 19% (from 0.36 to 0.43), 26% (from 0.39 to 0.49), and 21% (from 0.39 to 0.47), respectively for YFD, YGFW and YWT. These accuracy gains from BLUP to ssGBLUP were similar to the results of van der Werf et al. [47], who reported absolute gains in accuracy in meat and wool sheep between 5 and 15%. More recently, Gurman et al. [46] found accuracies of GEBVs for body weight traits of 0.55 in Australian Merino sheep with a relative increase of 0.16 from pedigree BLUP to ssGBLUP. Nel et al. [48], found ssGBLUP accuracies of 0.67 and 0.45 for fibre diameter and yearling liveweight in South-African Merino sheep, with a gain in accuracy of 8% and 22%. For YFD, we observed similar genomic prediction accuracy (0.69) for the Merino validation evaluated by themselves but higher gain (35%), meaning that our BLUP models were less accurate. For YWT, we found higher genomic prediction accuracy and gain from BLUP to ssGBLUP evaluations (0.66 and 47%), which could also be an effect of reference population size, which was smaller in their study (around 2000 animals with genotypes and phenotypes).

We also saw that the quality of predictions (bias, dispersion, accuracy) was more impacted across scenarios for the traits YGFW and YWT than for YFD. This can be explained by the difference in heritability between these traits, with YFD being highly heritable and the two other traits moderately heritable [7]. Indeed, studies have confirmed that for highly heritable traits, if direct phenotypes are available, additional information (genotypic or phenotypic information of the animal or related individuals) had very low influence on the prediction. Conversely, for traits with low or moderate heritability, additional information was more likely to improve the accuracy of predictions [30, 47, 49].

Regardless of the different models, the reference “All” yielded the best predictions: we did not observe significant biases, slopes were all close to one, and we observed the highest accuracies. More particularly, between the scenarios “D-D” and “D-All”, the GEBV accuracies increased of 0.07, 0.06 and 0.08 for YFD, YGFW and YWT, respectively. The combined evaluation was, therefore, beneficial, which was also observed for combined evaluations in dairy cattle [13, 16, 17] or dairy sheep [50]. Moreover, no deterioration of the quality of predictions of Merino validation animals was observed with the inclusion of Dohne and crossbred animals in the reference, whereas [15] found a negative effect of the inclusion of crossbred or other breeds in a purebred Merino evaluation. Oliveira et al. [51], working with meat sheep, found small gains in accuracy from separate to combined evaluation, and in some cases high bias.

Selecting animals more related to the Dohne, as in the scenario “C+D” (see Tables 1 and 2), was not beneficial. This is contrary to the findings of van den Berg et al. [16] in a combined genomic evaluation of several Australian dairy cattle breeds. The increase in reference population size, in the scenario “C+D”, was probably too small in comparison with the scenarios including the Merino reference population. Moreover, for this scenario the model “ssGBLUP-UPG” presented variable results for the traits YGFW and YWT, for all validation metrics. We can hypothesize that the Dohne crossbred individuals presented a high degree of diversity among them, which would have contributed to the variability of results observed from these models. We can assume that in the case of the “M+C” scenario, the very large Merino reference dominated the Crossbred, which is why we do not find these results inconsistent.

In terms of across-breed predictions, our study showed quite consistent results compared to the within breed predictions, even in the most “unfavorable” scenario (evaluation of Merino based on Dohne reference). This result differed from [50], who found that “across-[sub]populations” predictions in Lacaune dairy sheep breed were of low accuracy. This difference can be explained by the more distant genetic links between the [sub]populations studied in [50]. Similarly, across-flocks predictions can lead to highly variable accuracies depending on the level of relatedness between the reference and validation flocks in Merino breed; more specifically, it led to lower accuracies when the relatedness between reference and validations flocks was low [48]. This shows the important contribution of genetic links between the Australian Dohne and Merino breeds and the feasibility of a combined evaluation. In our study, a large proportion of the phenotypes were not associated with genotypes and were not identified as M, C or D phenotypes. Indeed, with the high level of linkage existing between Dohne Merino and Merino breeds, it is hard to differentiate them without genomic information. The phenotypes of non-genotyped individuals were therefore included in all our scenarios and most likely contributed to improving the quality of genomic predictions. Particularly in the scenarios involving the “D” reference, we can assume that the quality of the predictions would have been lower without these phenotypes.

When comparing UPG and metafounder models, we observed variable results for ssGBLUP-UPG models in some scenarios in comparison with metafounder models. For instance, we observed bias up to 4 genetic standard deviations for YGFW. We also found accuracies higher for crossbred or Dohne validation individuals than for Merino validations, in the case of references including Merino animals, which does not seem realistic. UPG models therefore appeared more unstable than metafounders in the case of ssGBLUP evaluations. This can be due to a lack of phenotypic information across time to estimate some UPGs, which can potentially be addressed in metafounder models through the a priori definition of covariances between groups. The importance of adapting the UPG definition to each trait according to the distribution of information as well as the heritability of the trait has already been highlighted by previous works that obtained very unstable results using ssGBLUP models with UPG [30]. Furthermore, in our study we redefined, compared to the MERINOSELECT evaluation, the way genetic groups were assigned, in order to improve ${\varvec{\Gamma}}$ estimation. It is possible that this redefinition may have negatively affected the estimation of some UPGs by introducing greater heterogeneity within groups, though this question is outside the scope of this study.

Overall, in the scenarios “All”, validation accuracies favored metafounder models, which was consistent with the conclusions of other studies [32, 33, 52, 53]. However, we observed slightly higher bias with metafounders in comparison to UPGs, in contrast with other studies [31, 34, 52]. Nevertheless, these biases were relatively low and we think that such bias would not hamper genetic gain. A further advantage of metafounder models is that they provide a more natural transition from pedigree-based to genomic evaluation [53], which is the expected pathway for the Dohne breed. Finally, we did not find significant differences between the metafounder method “GLS” or metafounder “Trend”. However, the “Trend” method estimates had the advantage of being less sensitive to lack of information for small groups, and is therefore the more robust method for practical application.

In practice, when a breed is transitioning from pedigree to genomic evaluation, even if the predictive benefits of the inclusion of genomics might be low initially, once genotypes are included, this often creates an incentive for breeders to genotype at a greater rate. This leads to an increase in the reference population size which will improve predictions for that breed. We can therefore expect an improvement in the efficiency of genomic selection a few generations after implementation.

Overall, our study suggests that the introduction of genomic information from the Dohne Merino breed into the MERINOSELECT evaluation, using metafounders to model missing pedigrees, is feasible and accurate.

## Conclusions

We found worthwhile genomic accuracies for Dohne genomic predictions, suggesting there is value in transitioning the Australian Dohne breed from pedigree-based to genomic selection. Our study also demonstrates that combining genomic evaluation of Dohne breed with the MERINOSELECT evaluation can enhance predictions accuracy for the breed, without impacting negatively on Merino predictions, resulting in a simpler, combined genomic prediction. Finally, metafounders may be a way to simplify and improve the genetic grouping in a combined evaluation, leading to enhanced predictions of breeding values.

## Supplementary Information

Supplementary Material 1. Figure S1. Split of the population into three groups (Merino, crossbred and Dohne Merino) based on PCA coordinates. Identification of purebred Dohne Merino, purebred Merino and Crossbred animals according to the PCA coordinates of their genotypes.

## Data Availability

The data are owned and managed by Meat and Livestock Australia and Sheep Genetics and access to the data can be negotiated by request.

## References

[1] Meat & Livestock Australia. Fast facts : Australia’s sheepmeat industry; 2023. https://www.mla.com.au/globalassets/mla-corporate/prices--markets/documents/trends--analysis/fast-facts--maps/mla_sheep-fast-facts-2023_300523.pdf. Accessed 6 Feb 2023.

[2] Swan AA, Brown DJ, van der Werf JHJ. Genetic variation within and between subpopulations of the Australian Merino breed. Anim Prod Sci. 2015;56:87–94.

[3] Gurman PM, Swan AA, Boerner V. Use of genomic data to determine breed composition of Australian sheep. Proc Assoc Adv Anim Breed Genet. 2017;22:341–4.

[4] Swan AA, Banks RG, Brown DJ, Chandler HR. An update on genetic progress in the Australian sheep industry. Proc Assoc Adv Anim Breed Genet. 2017;22:365–8.

[5] Brown DJ, Swan AA, Boerner V, Li L, Gurman PM, McMillan AJ. Single-Step Genetic Evaluations in the Australian Sheep Industry. In: Proceedings of the 11th world congress on genetics applied to livestock production: 11–16 February 2018; Auckland; 2018.

[6] Meat & Livestock Australia. The transitioning Australian sheep flock—where have we come from ? Where are we now ? Meat & Livestock Australia; 2015. https://www.mla.com.au/prices-markets/market-news/2015/the-transitioning-australian-sheep-flock--where-have-we-come-from-where-are-we-now/. Accessed 6 Feb 2023.

[7] Mortimer SI, Hatcher S, Fogarty NM, van der Werf JHJ, Brown DJ, Swan AA, et al. Genetic parameters for wool traits, live weight, and ultrasound carcass traits in Merino sheep. J Anim Sci. 2017;95:1879–91. 28726993 10.2527/jas.2016.1234

[8] Dominik S, Swan AA. Genetic and phenotypic parameters for reproduction, production and bodyweight traits in Australian fine-wool Merino sheep. Anim Prod Sci. 2016;58:207–12.

[9] Cloete SWP, Schoeman SJ, Coetzee J, de Morris JV. Genetic variances for liveweight and fleece traits in Merino, Dohne Merino and South African Meat Merino sheep. Aust J Exp Agric. 2001;41:145–53.

[10] Casey AE, Wilson BCD. Dohne Ram breeders manual. https://dohne.com.au/wp-content/uploads/2016/08/THE-DOHNE-IN-AUSTRALIA.pdf. Accessed 6 Feb 2023.

[11] Sheep genetics. Dohne Index—a ram breeder’s guide. Sheep genetics; 2022. https://www.sheepgenetics.org.au/globalassets/sheep-genetics/getting-started/sheep-genetics---asbvs-and-indexes/dohne-indexes-2022.pdf. Accessed 6 Feb 2023.

[12] Nel C, Gurman P, Swan A, van der Werf J, Snyman M, Dzama K, et al. The genomic structure of isolation across breed, country and strain for important South African and Australian sheep populations. BMC Genomics. 2022;23:23. 34983377 10.1186/s12864-021-08020-3 PMC8725491

[13] Brøndum RF, Rius-Vilarrasa E, Strandén I, Su G, Guldbrandtsen B, Fikse WF, et al. Reliabilities of genomic prediction using combined reference data of the Nordic Red dairy cattle populations. J Dairy Sci. 2011;94:4700–7. 21854944 10.3168/jds.2010-3765

[14] Jónás D, Ducrocq V, Fritz S, Baur A, Sanchez M-P, Croiseau P. Genomic evaluation of regional dairy cattle breeds in single-breed and multibreed contexts. J Anim Breed Genet. 2017;134:3–13. 27917542 10.1111/jbg.12249

[15] Moghaddar N, Swan AA, van der Werf JH. Comparing genomic prediction accuracy from purebred, crossbred and combined purebred and crossbred reference populations in sheep. Genet Sel Evol. 2014;46:58. 25927315 10.1186/s12711-014-0058-4 PMC4180850

[16] van den Berg I, MacLeod IM, Reich CM, Breen EJ, Pryce JE. Optimizing genomic prediction for Australian Red dairy cattle. J Dairy Sci. 2020;103:6276–98. 32331891 10.3168/jds.2019-17914

[17] Zhou L, Heringstad B, Su G, Guldbrandtsen B, Meuwissen THE, Svendsen M, et al. Genomic predictions based on a joint reference population for the Nordic Red cattle breeds. J Dairy Sci. 2014;97:4485–96. 24792791 10.3168/jds.2013-7580

[18] de las Heras-Saldana S, Gurman PM, Swan AA, Brown DJ. Genetic parameters and lambda values for post-weaning production traits in Merino sheep. Proc Assoc Adv Anim Breed Genet. 2023;25:318–21.

[19] Aguilar I, Misztal I, Tsuruta S, Legarra A, Huiyu Wang. PREGSF90—POSTGSF90: computational tools for the implementation of single-step genomic selection and genome-wide association with ungenotyped individuals in BLUPF90 programs. In: Proceedings of the 10th world congress on genetics applied to livestock production: 18–22 August 2014; Vancouver. 2014.

[20] Browning BL, Tian X, Zhou Y, Browning SR. Fast two-stage phasing of large-scale sequence data. Am J Hum Genet. 2021;108:1880–90. 34478634 10.1016/j.ajhg.2021.08.005 PMC8551421

[21] Baglama J, Reichel L, Lewis BW. Fast truncated singular value decomposition and principal components analysis for large dense and sparse matrices; 2022. https://CRAN.R-project.org/package=irlba. Accessed 6 Feb 2023.

[22] Hudson RR, Slatkin M, Maddison WP. Estimation of levels of gene flow from DNA sequence data. Genetics. 1992;132:583–9. 1427045 10.1093/genetics/132.2.583 PMC1205159

[23] Bhatia G, Patterson N, Sankararaman S, Price AL. Estimating and interpreting *F*_ST_ : the impact of rare variants. Genome Res. 2013;23:1514–21. 23861382 10.1101/gr.154831.113 PMC3759727

[24] VanRaden PM, Van Tassell CP, Wiggans GR, Sonstegard TS, Schnabel RD, Taylor JF, et al. Invited review: reliability of genomic predictions for North American Holstein bulls. J Dairy Sci. 2009;92:16–24. 19109259 10.3168/jds.2008-1514

[25] Hayes BJ, Bowman PJ, Chamberlain AJ, Goddard ME. Invited review: genomic selection in dairy cattle: progress and challenges. J Dairy Sci. 2009;92:433–43. 19164653 10.3168/jds.2008-1646

[26] Legarra A, Reverter A. Semi-parametric estimates of population accuracy and bias of predictions of breeding values and future phenotypes using the LR method. Genet Sel Evol. 2018;50:53. 30400768 10.1186/s12711-018-0426-6 PMC6219059

[27] Quaas RL. Additive genetic model with groups and relationships. J Dairy Sci. 1988;71:1338–45.

[28] Legarra A, Christensen OF, Vitezica ZG, Aguilar I, Misztal I. Ancestral relationships using metafounders: finite ancestral populations and across population relationships. Genetics. 2015;200:455–68. 25873631 10.1534/genetics.115.177014 PMC4492372

[29] Meyer K, Swan AA. “Metafounders” to model base populations in genomic evaluation for multi-breed sheep data. Proc Assoc Adv Anim Breed Genet. 2019;23:27–30.

[30] Bradford HL, Masuda Y, VanRaden PM, Legarra A, Misztal I. Modeling missing pedigree in single-step genomic BLUP. J Dairy Sci. 2019;102:2336–46. 30638995 10.3168/jds.2018-15434

[31] Kluska S, Masuda Y, Ferraz JBS, Tsuruta S, Eler JP, Baldi F, et al. Metafounders may reduce bias in composite cattle genomic predictions. Front Genet. 2021;12: 678587. 34490031 10.3389/fgene.2021.678587 PMC8417888

[32] Kudinov AA, Koivula M, Aamand GP, Strandén I, Mäntysaari EA. Single-step genomic BLUP with many metafounders. Front Genet. 2022;13:1012205. 36479243 10.3389/fgene.2022.1012205 PMC9721289

[33] Kudinov AA, Mäntysaari EA, Aamand GP, Uimari P, Strandén I. Metafounder approach for single-step genomic evaluations of Red Dairy cattle. J Dairy Sci. 2020;103:6299–310. 32418688 10.3168/jds.2019-17483

[34] Garcia-Baccino CA, Legarra A, Christensen OF, Misztal I, Pocrnic I, Vitezica ZG, et al. Metafounders are related to F_st_ fixation indices and reduce bias in single-step genomic evaluations. Genet Sel Evol. 2017;49:34. 28283016 10.1186/s12711-017-0309-2 PMC5439149

[35] Sorensen DA, Kennedy BW. The use of the relationship matrix to account for genetic drift variance in the analysis of genetic experiments. Theor Appl Genet. 1983;66:217–20. 24263919 10.1007/BF00251147

[36] Legarra A, Bermann M, Mei Q, Christensen OF. Redefining and interpreting genomic relationships of metafounders. Genet Sel Evol. 2024; 56(1):34–40. 10.1186/s12711-024-00891-w PMC11536960 38698373

[37] Gilmour AR, Cullis BR, Welham SJ, International V, Hempstead H, Thompson R. ASReml-SA. User guide release 4.2 functional specification. Hemel Hempstead: VSN International Ltd; 2022.

[38] Brown DJ, Huisman AE, Swan AA, Graser H-U, Woolaston RR, Ball AJ, et al. Genetic evaluation for the Australien sheep industry. Proc Assoc Adv Anim Breed Genet. 2007;17:187–94.

[39] Brown DJ, Tier B, Reverter A, Banks R, Graser HU. OVIS: A multiple trait breeding value estimation program for genetic evaluation of sheep. Wool Technol Sheep Breed. 2000;48:285–97.

[40] Brown DJ, Reverter A. A comparison of methods to pre-adjust data for systematic environmental effects in genetic evaluation of sheep. Livest Prod Sci. 2002;75:281–91.

[41] Brown DJ, Atkins K, Huisman AE. Expression of body weight, fleece weight and fibre diameter in across flock genetic evaluation. Proc Assoc Adv Anim Breed Genet. 2005;16:84–87.

[42] Masuda Y, VanRaden PM, Tsuruta S, Lourenco DAL, Misztal I. Invited review: unknown-parent groups and metafounders in single-step genomic BLUP. J Dairy Sci. 2022;105:923–39. 34799109 10.3168/jds.2021-20293

[43] VanRaden PM. Efficient methods to compute genomic predictions. J Dairy Sci. 2008;91:4414–23. 18946147 10.3168/jds.2007-0980

[44] Lourenco D, Tsuruta S, Aguilar I, Masuda Y, Bermann M, Legarra A, et al. 366. Recent updates in the BLUPF90 software suite. In: Proceedings of the 12th world congress on genetics applied to livestock production: 3–8 July 2022; Rotterdam; 2022.

[45] Dzomba EF, Van Der Nest MA, Mthembu JNT, Soma P, Snyman MA, Chimonyo M, et al. Selection signature analysis and genome-wide divergence of South African Merino breeds from their founders. Front Genet. 2023;13: 932272. 36685923 10.3389/fgene.2022.932272 PMC9847500

[46] Gurman PM, Swan AA, Boerner V, Brown DJ. Cross-validation of single step BLUP applied to terminal sire sheep in Australia. In: Proceedings of the 11th world congress on genetics applied to livestock production: 11–16 February 2018; Auckland; 2018.

[47] van der Werf JHJ, Banks RG, Clark SA, Lee SJ, Daetwyler HD, Hayes BJ, et al. Genomic selection in sheep breeding programs. In: Proceedings the 10th world congress of genetics applied to livestock production: 18–22 august 2014; Vancouver; 2014.

[48] Nel C, Gurman P, Swan A, van der Werf J, Snyman M, Dzama K, et al. Including genomic information in the genetic evaluation of production and reproduction traits in South African Merino sheep. J Anim Breed Genet. 2024;141:65–82. 37787180 10.1111/jbg.12826

[49] Hidalgo J, Lourenco D, Tsuruta S, Bermann M, Breen V, Herring W, et al. Efficient ways to combine data from broiler and layer chickens to account for sequential genomic selection. J Anim Sci. 2023;101:skad177. 37249185 10.1093/jas/skad177 PMC10276640

[50] Wicki M, Raoul J, Legarra A. Effect of subdivision of the Lacaune dairy sheep breed on the accuracy of genomic prediction. J Dairy Sci. 2023;106:5570–81. 37349212 10.3168/jds.2022-23114

[51] Oliveira HRD, McEwan JC, Jakobsen JH, Blichfeldt T, Meuwissen THE, Pickering NK, et al. Across-country genomic predictions in Norwegian and New Zealand Composite sheep populations with similar development history. J Anim Breed Genet. 2022;139:1–12. 34418183 10.1111/jbg.12642

[52] Macedo FL, Astruc JM, Meuwissen THE, Legarra A. Removing data and using metafounders alleviates biases for all traits in Lacaune dairy sheep predictions. J Dairy Sci. 2022;105:2439–52. 35033343 10.3168/jds.2021-20860

[53] Macedo FL, Christensen OF, Astruc J-M, Aguilar I, Masuda Y, Legarra A. Bias and accuracy of dairy sheep evaluations using BLUP and SSGBLUP with metafounders and unknown parent groups. Genet Sel Evol. 2020;52:47. 32787772 10.1186/s12711-020-00567-1 PMC7425573

