# Supplementary figures and images for: Combined genomic evaluation of Merino and Dohne Merino Australian sheep populations
Source: Genet Sel Evol. 2024 Sep 30;56:69. doi: 10.1186/s12711-024-00934-2 (PMC11440750; doi:10.1186/s12711-024-00934-2)

Split of population according to PCA coordinates

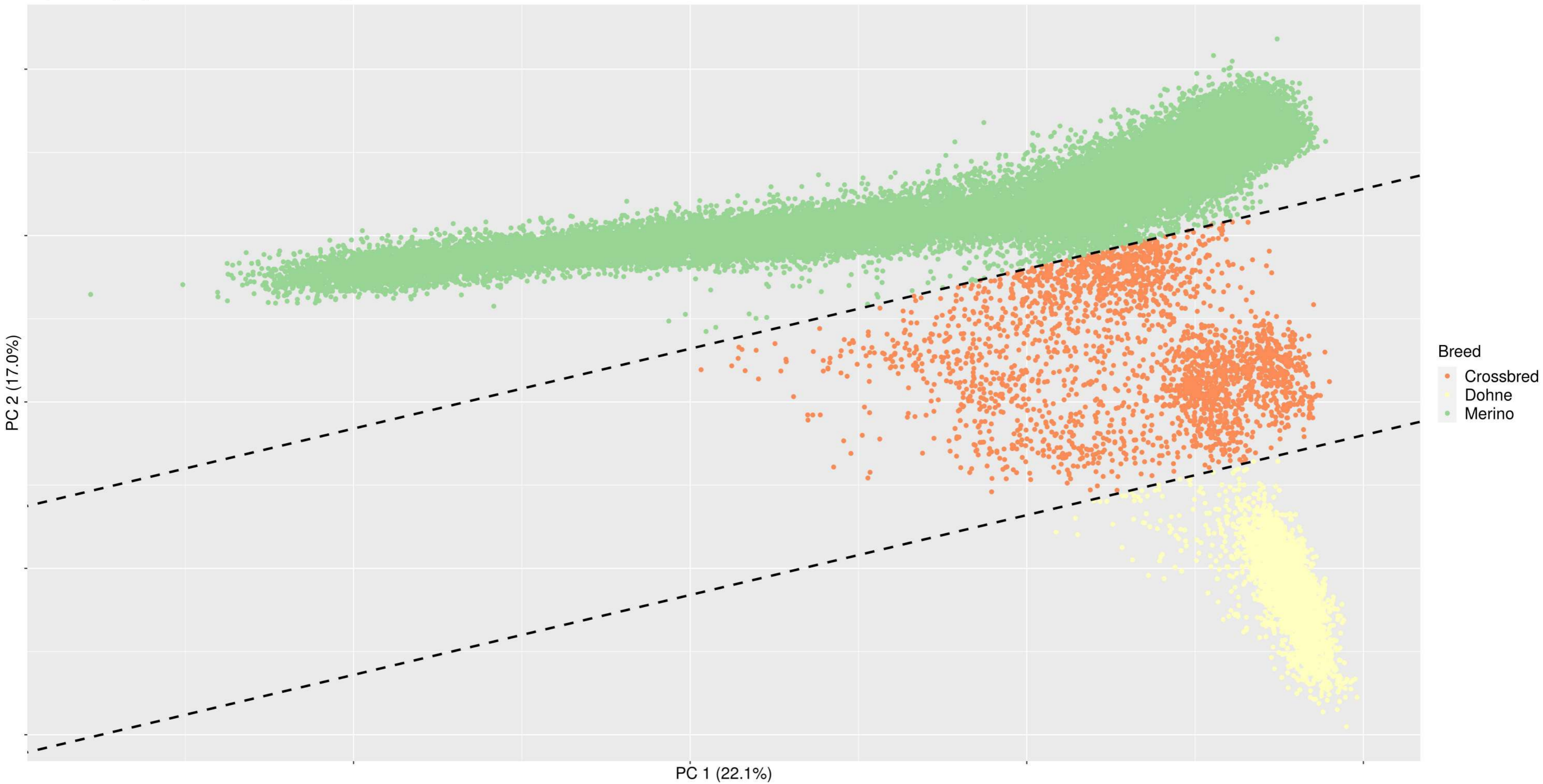

Supplement: Supplementary file 1 — Supplementary Material 1. Figure S1. Split of the population into three groups (Merino, crossbred and Dohne Merino) based on PCA coordinates. Identification of purebred Dohne Merino, purebred Merino and Crossbred animals according to the PCA coordinates of their genotypes. [file 12711_2024_934_MOESM1_ESM.pdf]
